# Flocking Behaviors under Hierarchical Leadership of Thermodynamic Cucker–Smale Particles with Multiplicative White Noise and Perturbation

**DOI:** 10.3390/e25030417

**Published:** 2023-02-25

**Authors:** Shuobing Yang, Yinghua Jin, Aihua Hu, Yipeng Shao

**Affiliations:** School of Science, Jiangnan University, Wuxi 214122, China

**Keywords:** thermodynamic Cucker–Smale model, flocking, hierarchical leadership, white noise

## Abstract

The thermodynamic Cucker–Smale model (TCS model) describes dynamic consistency caused by different temperatures between multi-agent particles. This paper studies the flocking behaviors of the TCS model with multiplicative white noise under hierarchical leadership. First, we introduce the corresponding model of two particles. Then, by using mathematical induction and considering the properties of differential functions, it is proved that, under certain conditions, the group can achieve flocking. Finally, we verify the conclusion through numerical simulation results. Similarly, this paper studies the above model with perturbation functions.

## 1. Introduction

Classical flocking dynamics in nature include the coordinated flight of birds, the vortices of fish, the collective migration of ant colonies, and the growth and self-organizing activation of cells, etc. [1,2]. Rich and highly coordinated flocking behaviors emerge in systems composed of interconnected and constantly moving individuals. The main purpose of flocking research is to reveal dynamic behaviors of complex systems and guide engineering applications such as UAV flight and robot formation.

Flocking describes a phenomenon in which self-driven particles are connected by simple rules and organized from a disordered state to ordered motion. Vicsek [3] described the formation mechanism of flocking behavior by means of numerical experiments. Jadbabaie [4] strictly proved the correctness of the above numerical experiments based on certain assumptions from the view point of mathematics. Since then, many mathematical models have emerged to study the consistency and flocking behavior of multi-agent systems [5,6,7]. In 2007, Cucker and Smale [8] proposed the Cucker–Smale model (CS model) on the basis of the Vicsek model. The dynamic equation of the *i*-th particle is as follows

(1) $$\begin{array}{c} dx_{i}=v_{i}dt\\ dv_{i}=\frac{1}{N}\sum_{1\leq k\leq N}\phi_{ik}\left(v_{k}-v_{i}\right)dt \end{array}$$

where $\phi_{ik}=1/{\left(1+{∥x_{i}\left(t\right)-x_{k}\left(t\right)∥}^{2}\right)}^{\beta }$ is the communication weight. When β≤0.5, an unconditional flocking of the CS model is achieved. In this model, there is no constraint that velocities must be the same. Furthermore, the communication weight is related to the absolute distance between the *i*-th particle and its neighbor. It is consistent with the actual situation. Many scholars have carried out research on the CS model [9,10,11,12].

Shen [13] studied the CS system with asymmetric interaction structure, which is the CS system under hierarchy. Each particle is only affected by the leadership set. When β≤0.5, the continuous system can achieve unconditional cluster motion.

Ha [14] presented a thermodynamically consistent particle (TCP) model based on the theory of multi-temperature mixing of fluids in the case of spatially homogeneous processes. The proposed thermodynamic model includes the flocking model of the CS type as its isothermal approximation. Then, Ha [15] proposed a new type of CS model, called a thermodynamic Cucker–Smale model (TCS model). It describes a phenomenon caused by different internal temperature variations between particles in the gas mixture system. If the initial temperature is homogeneous ($\theta_{i}^{0}=1$), the system is reduced to the CS model. The TCS system is derived by the entropy theory on the basis of the traditional dynamic system, which is a fusion of thermodynamics and complex networks. The TCS model has a noise term not only in the velocity differential equation but also in the temperature equation. The estimation of this asymmetric network has much broader real-world applications. Many scholars have conducted in-depth research on the TCS system [16,17,18].

Most modeling of flocking systems only considers the dynamics of the same particles. In real scenarios, however, we need to study the flocking of different particles. Firstly, our TCS model can better simulate the temperature variation of the particles in the system in a more realistic way. The TCS model describes the temperature variation of the particles and explores the internal energy, while the traditional CS model only considers the mechanical motion of the particles. Secondly, we study the TCS system with perturbations to describe the flocking behavior of systems composed of different particles. The active particles discussed in our system are not identical and their motion has some degrees of freedom. Moreover, the establishment of leaders is a common tool in formation control, so the TCS model is studied under hierarchy to simulate the situation. Finally, the inclusion of white noise can better model the effect of environmental factors on the system. Based on this, this paper investigates the flocking behavior of the TCS model with multiplicative white noise under a hierarchical leader. The particles considered in the system are not identical and perturbation terms are introduced into the velocity variation of the particles, allowing a degree of flexibility for each particle. It is shown that the system can satisfy flocking under certain conditions. This conclusion is verified by numerical simulation results.

## 2. System Descriptions and Main Results

**Definition** **1** ([13]). *A group $\left\{1,\cdots ,N\right\}$ is said to be under a hierarchical leadership if, for all $x\in R^{d}$, the adjacency matrix $A_{x}=\left(a_{ij}\left(x\right)\right)$ satisfies (1) $a_{ij}\neq 0\left(j<i\right)$; (2) for all i>1, the set $L\left(i\right)=\left\{j\left|a_{ij}>\right.0\right\}\neq \emptyset$. $L\left(i\right)$ is the leading set of the i-th particle.*

Our model is described as follows

(2) $$\begin{array}{c} dx_{i}=v_{i}dt\\ dv_{i}=\lambda \sum_{k\in L\left(i\right)}\phi_{ik}\left(\frac{v_{k}}{\theta_{k}}-\frac{v_{i}}{\theta_{i}}\right)dt+\sigma_{i}\sum_{k\in L\left(i\right)}\phi_{ik}\left(v_{k}-v_{i}\right)dW_{i}^{t}\\ d\theta_{i}=\mu \sum_{k\in L\left(i\right)}\zeta_{ik}\left(\frac{1}{\theta_{i}}-\frac{1}{\theta_{k}}\right)dt \end{array}$$

where λ,μ>0 and $\sigma_{i}\in R$ are coupling strengths and noise intensity coefficient $W_{i}^{t}$ is one-dimensional Brownian motion. We consider the white Gauss noises [19,20] which are independently identically distributed.

$$E\left(dW_{i}^{t}\right)=0,V\left(dW_{i}^{t}dW_{j}^{t^{*}}\right)=\delta_{ij}\delta \left(t-t_{*}\right)$$

where $E\left(\cdot \right),V\left(\cdot \right)$ are the expectation and variance of random variables, respectively. Furthermore, $\phi_{ik},\zeta_{ik}$ are the inter-individual connection weight functions [21].

$$\begin{array}{cc} \phi_{ik} & =1/{\left(1+{∥x_{i}\left(t\right)-x_{k}\left(t\right)∥}^{2}\right)}^{\beta }\left(\beta \leq 0.5\right)\\ \zeta_{ik} & =1/{\left(1+{∥x_{i}\left(t\right)-x_{k}\left(t\right)∥}^{2}\right)}^{\beta }\left(\beta \leq 0.5\right) \end{array}$$

Furthermore, we study System (2) with disturbance functions of time $f_{i}\left(t\right),t\geq 0$. The TCS model is turned into

(3) $$\begin{array}{c} dx_{i}=v_{i}dt\\ dv_{i}=\lambda \sum_{k\in L\left(i\right)}\phi_{ik}\left(\frac{v_{k}}{\theta_{k}}-\frac{v_{i}}{\theta_{i}}\right)dt+f_{i}dt+\sigma_{i}\sum_{k\in L\left(i\right)}\phi_{ik}\left(v_{k}-v_{i}\right)dW_{i}^{t}\\ d\theta_{i}=\mu \sum_{k\in L\left(i\right)}\zeta_{ik}\left(\frac{1}{\theta_{i}}-\frac{1}{\theta_{k}}\right)dt \end{array}$$

**Definition** **2** ([14]). *A group is said to be flocking if, for all $x,v\in R^{d}$, θ∈R satisfy (1) $\sup E∥x_{i}-x_{k}∥<\infty$ (2) $\lim_{t\to \infty }E∥v_{i}-v_{k}∥=0$ (3) $\lim_{t\to \infty }E∥\theta_{i}-\theta_{k}∥=0$.*

The flocking behaviors of systems are discussed under the above definition. The main results are as follows. For System (2), we have

**Theorem** **1.** *We suppose that $\left\{1,2,\cdots ,N\right\}$ is a hierarchical group. Particle 1 is the global leader and moves at a constant temperature. For t→∞, if β<1/2, the temperatures of System (2) have asymptotic consistency.*

**Theorem** **2.** *We suppose that $\left\{1,2,\cdots ,N\right\}$ is a hierarchical group. Particle 1 is the global leader and moves at a constant speed. If, for $t\to \infty ,\beta <1/2,\sigma <\sqrt{\frac{2\lambda }{\theta_{M}^{0}}-1},\sigma :=\max\sigma_{i}\left(i=1,2,\cdots ,N\right)$, System (2) can achieve flocking.*

For System (3), we have

**Theorem** **3.** *We suppose that $\left\{1,2,\cdots ,N\right\}$ is a hierarchical group. System (3) satisfies $\int_{0}^{\infty }$ $∥f_{i}\left(t\right)∥dt$ <∞ and $E∥f_{i}-f_{j}∥\leq qe^{-pt}$. Particle 1 is the global leader and the change in velocity only depends on $f_{1}$. If, for $t\to \infty ,\beta <1/2,\sigma <\sqrt{\frac{2\lambda }{\theta_{M}^{0}}-1},\sigma :=\max\sigma_{i}\left(i=1,2,\cdots ,N\right)$, System (3) can achieve flocking.*

## 3. Result Proof of System (2)

### 3.1. Proof of Theorem 1

**Lemma** **1.** *Let ${\left(x_{i},v_{i},\theta_{i}\right)}_{i=1}^{N}$ be the solution of the TCS system. Temperatures have the minimum value $\theta_{i}\left(t\right)\geq \theta_{\min},i=1,2,\cdots ,N$.*

**Proof.**  $\varepsilon \in \left(0,\theta_{\min}\right)$ is given. We defined a set

(4) $$S:=\left\{T>0\left|\theta_{i}\left(t\right)>\theta_{\min}-\varepsilon ,t\in \left[0,T\right],i=1,\ldots ,N\right.\right\}$$

From the continuity of $\theta_{i}$ and the definition of $\theta_{\min}$, it follows that S≠∅ and then supS=∞ is proved by reduction to absurdity.

We consider $T^{*}:=\sup S<\infty$; hence there exists $1\leq i^{*}\leq N$ obtaining $\theta_{i^{*}}\left(T^{*}\right)=\theta_{min}-\varepsilon$. For $0<t<T^{*}$, since ζ is a monotone subtracting function and $\zeta \left(0\right)=1$, we have

(5) $$\begin{array}{cc} \frac{d\theta_{i^{*}}}{dt} & =\mu \sum_{k\in L\left(i^{*}\right)}\zeta_{i^{*}k}\left(\frac{1}{\theta_{i^{*}}}-\frac{1}{\theta_{k}}\right)\\ & >\mu \sum_{k\in L\left(i^{*}\right)}\zeta_{i^{*}k}\left(\frac{1}{\theta_{i^{*}}}-\frac{1}{\theta_{min}-\varepsilon }\right)=\frac{\theta_{min}-\varepsilon -\theta_{i^{*}}}{\theta_{i^{*}}\left(\theta_{min}-\varepsilon \right)}\mu \sum_{k\in L\left(i^{*}\right)}\zeta_{i^{*}k} \end{array}$$

Because ζ is a monotone decreasing function, $\zeta \left(0\right)=1$ and $\theta_{i^{*}}>\theta_{min}-\varepsilon$, there is $\frac{\theta_{min}-\varepsilon -\theta_{i^{*}}}{\theta_{i^{*}}\left(\theta_{min}-\varepsilon \right)}<0$, such that

(6) $$\frac{d\theta_{i^{*}}}{dt}\geq \frac{\theta_{min}-\varepsilon -\theta_{i^{*}}}{\theta_{i^{*}}\left(\theta_{min}-\varepsilon \right)}\mu i^{*}\zeta \left(0\right)=\frac{\mu i^{*}\left(\theta_{min}-\varepsilon -\theta_{i^{*}}\right)}{\theta_{i^{*}}\left(\theta_{min}-\varepsilon \right)}$$

It yields

(7) $$\frac{\left(\theta_{min}-\varepsilon \right)\theta_{i^{*}}}{\theta_{i^{*}}-\left(\theta_{min}-\varepsilon \right)}\frac{d\theta_{i^{*}}}{dt}>-\mu i^{*}$$

We integral (7) to *t*

(8) $$\left(\theta_{min}-\varepsilon \right)\left(\theta_{i^{*}}\left(t\right)-\theta_{i^{*}}\left(0\right)\right)+{\left(\theta_{min}-\varepsilon \right)}^{2}\log\frac{\theta_{i^{*}}\left(t\right)-\left(\theta_{min}-\varepsilon \right)}{\theta_{i^{*}}\left(0\right)-\left(\theta_{min}-\varepsilon \right)}\geq -\mu i^{*}t,0<t<T^{*}$$

When $t\to T^{*},\theta_{i^{*}}\to \theta_{min}-\varepsilon$, the left value of (8) tends to −∞. It yields a conflict, which means $T^{*}=\infty$. Therefore, for any $\varepsilon \in \left(0,\theta_{\min}\right)$, we have

(9) $$\theta_{i}\left(t\right)>\theta_{min}-\varepsilon ,t\in \left[0,\infty \right),i=1,\cdots ,N$$

According to the arbitrariness of ε,

(10) $$\theta_{i}\left(t\right)\geq \theta_{min},t\in \left[0,\infty \right),i=1,\cdots ,N$$

It is obvious that the minimum value $\theta_{m}^{0}:=\theta_{\min}=\min_{k}\theta_{k}\left(0\right)$. □

**Remark** **1.** *We explain the maximum situation $\theta_{M}^{0}:=\theta_{\max}=\max_{k}\theta_{k}\left(0\right)$, whose proof is the same as the minimum part.*

**Remark** **2.** *According to $E∥\theta_{\min}∥\leq E∥\theta_{i}∥\leq E∥\theta_{\max}∥$, $E∥\theta_{i}-\theta_{j}∥\leq E∥\theta_{i}∥+E∥\theta_{j}∥\leq D$, where D>0 is a constant.*

**Lemma** **2.** *If particle 1 is the global leader and moves at a constant speed, for any t>0, $E∥v_{i}\left(t\right)∥\leq D\left(1\leq i\leq N\right)$, where D>0 is a constant.*

**Proof.**  First, we prove $E∥v_{2}\left(t\right)∥$ is bounded by reduction to absurdity. If $E∥v_{2}\left(t\right)∥$ is unbounded, there exists a component $j\left(1\leq j\leq d\right)$ which satisfies $\lim_{t\to \infty }Ev_{2}^{j}\left(t\right)=\infty$. Integrate the second equation of the system and calculate the expected result

(11) $$v_{2}^{j}\left(0\right)=Ev_{2}^{j}\left(t\right)+\lambda \int_{0}^{t}E\phi_{21}\left(\frac{v_{1}^{j}\left(0\right)}{\theta_{1}\left(0\right)}-\frac{v_{2}^{j}\left(s\right)}{\theta_{2}\left(s\right)}\right)\mathrm{ds}$$

Since $\theta_{\min}<\theta_{2}\left(s\right),\theta_{1}\left(0\right)<\theta_{\max}$, (11) implies $v_{2}^{j}\left(0\right)=\infty$. A contradiction appears, which means $E∥v_{2}\left(t\right)∥$ is bounded.

We assume that $E∥v_{2}\left(t\right)∥,\cdots ,E∥v_{l-1}\left(t\right)∥\left(l-1\geq 3\right)$ are bounded. The presence of D≥0 makes $\max_{1\leq i\leq l-1}∥v_{i}\left(t\right)∥\leq D$. A mathematical induction method is used to prove $E∥v_{l}\left(t\right)∥$ is bounded.

Assuming that $E∥v_{l}\left(t\right)∥$ is unbounded, there exists a component $j\left(1\leq j\leq d\right)$ which satisfies $\lim_{t\to \infty }Ev_{l}^{j}\left(t\right)=\infty$. Integrate the equation and calculate the expected result

(12) $$v_{l}^{j}\left(0\right)=Ev_{l}^{j}\left(t\right)+\lambda \int_{0}^{t}\sum_{k\in L\left(l\right)}E\phi_{lk}\left(\frac{v_{l}^{j}\left(s\right)}{\theta_{l}\left(s\right)}-\frac{v_{k}^{j}\left(s\right)}{\theta_{k}\left(s\right)}\right)\mathrm{ds}$$

Since $\theta_{\min}<\theta_{l}\left(s\right),\theta_{k}\left(s\right)<\theta_{\max}$, (12) implies $v_{l}^{j}\left(0\right)=\infty$. A contradiction appears, which means $E∥v_{l}\left(t\right)∥$ is bounded. □

Because of the norm inequality $E∥v_{i}-v_{j}∥\leq E∥v_{i}∥+E∥v_{j}∥$, there is a $D_{0}$ satisfying $E∥v_{i}-v_{j}∥\leq D_{0}$.

**Lemma** **3.** *We consider a hierarchical population of two individuals, corresponding to $\theta_{2}-\theta_{1}$, satisfying the following equation*

(13) $$d\theta =\mu \zeta \left(\frac{1}{\theta_{2}}-\frac{1}{\theta_{1}}\right)dt+\xi dt$$

*including $\zeta \left(∥x∥\right)\in R$*

(1)
$1\geq \zeta \left(x,t\right)={\left(1+{∥x\left(t\right)∥}^{2}\right)}^{-\beta }>0$

(2)
$E∥\xi ∥\leq qe^{-pt}\left(p,q>0\right)$

*There are P,Q>0, for all t>0, bringing $E∥\theta \left(t\right)∥\leq Qe^{-Pt^{\left(1-2\beta \right)/2}}$ into existence.*

**Proof.**  From (13), we have

(14) $$d\theta^{2}=2\theta d\theta =\left(-\frac{\mu \zeta }{\theta_{2}\theta_{1}}\theta^{2}+2\theta \xi \right)dt$$

From Lemma 1, there is a nonnegative constant *D* causing $E∥\theta ∥\leq D$. We take advantage of the property of expectation

(15) $$2E\left(\theta \xi \right)\leq 2E∥\theta ∥E∥\xi ∥\leq 2Dqe^{-pt}$$

For $E∥x\left(t\right)∥=x\left(0\right)+\int_{0}^{t}E∥v\left(s\right)∥ds\leq x\left(0\right)+D_{0}t$, when $t>T_{1}$, there exist $T_{1}>0,m_{1}>0$ satisfying

(16) $$\left[{\left(1+{∥x\left(t\right)∥}^{2}\right)}^{-\beta }\right]\geq m_{1}{∥x\left(t\right)∥}^{-2\beta }$$

Therefore,

(17) $$E\left[\zeta \left(t\right)\right]=E\left[{\left(1+{∥x\left(t\right)∥}^{2}\right)}^{-\beta }\right]\geq m_{1}E\left[{∥x\left(t\right)∥}^{-2\beta }\right]\geq m_{1}{\left[x\left(0\right)+D_{0}t\right]}^{-2\beta }$$

When $t>x\left(0\right)/D_{0}$, similarly to $x\left(0\right)<D_{0}t$, we have $m_{1}{\left[x\left(0\right)+D_{0}t\right]}^{-2\beta }>m_{1}{\left(2D_{0}t\right)}^{-2\beta }$. There exists $T=\max\left(T_{1},x\left(0\right)/D\right)$, when t>T, such that $E\left(\zeta \right)>mt^{-2\beta }\left(m=m_{1}{\left(2D_{0}\right)}^{-2\beta }\right)$. Since $\zeta \left(t\right)<1$, there are $\zeta^{2}\left(t\right)<\zeta \left(t\right)$ and $E\left[\zeta^{2}\left(t\right)\right]<E\left[\zeta \left(t\right)\right]$. For $\theta_{1},\theta_{2}<\theta_{M}^{0}$,

(18) $$E\left(-\frac{\mu \zeta }{\theta_{2}\theta_{1}}\right)\leq E\left(-\frac{\mu \zeta }{{\left(\theta_{M}^{0}\right)}^{2}}\right)=-\frac{\mu }{{\left(\theta_{M}^{0}\right)}^{2}}E\left(\zeta \right)<-\frac{\mu m}{{\left(\theta_{M}^{0}\right)}^{2}}t^{-2\beta }:=b\left(t\right)$$

From (14)–(18), by defining $d\Theta =\left(-b\left(t\right)V+2Dqe^{-pt}\right)dt,\Theta \left(0\right)=\theta^{2}\left(0\right)$, we obtain

(19) $$\begin{array}{cc} E\left(\Theta \right) & =e^{-\int b\left(t\right)dt}\left(\int 2Dqe^{-pt}e^{\int b\left(t\right)dt}dt+C\right)\\ & =\theta^{2}\left(0\right)e^{-\frac{\mu m}{{\left(\theta_{M}^{0}\right)}^{2}\left(1-2\beta \right)}t^{1-2\beta }}\left(\int 2Dqe^{-pt}e^{\frac{\mu m}{{\left(\theta_{M}^{0}\right)}^{2}\left(1-2\beta \right)}t^{1-2\beta }}dt+1\right) \end{array}$$

There are P,Q satisfying $E\left(\Theta \right)\leq Qe^{-Pt^{1-2\beta }}$. According to the comparison theorem, when t>T,

(20) $$\lim_{t\to \infty }E\left(\theta^{2}\right)\leq \lim_{t\to \infty }E\left(\Theta \right)\leq Qe^{-Pt^{1-2\beta }}$$

□

Next, we give the proof of Theorem 1.

**Proof.**  (Proof of Theorem 1)

The conclusion is proved to be valid for a subpopulation $\left\{1,2\right\}$. According to the definition of hierarchical groups, $L\left(2\right)=1,\zeta_{21}>0$, and the thermostatic movement of agent1, the following equation can be known

(21) $$d\theta_{1}=0,d\theta_{2}=\lambda \zeta_{21}\left(\frac{1}{\theta_{2}}-\frac{1}{\theta_{1}}\right)dt$$

We consider

(22) $$\theta =\theta_{2}-\theta_{1}$$

It is available that

(23) $$d\theta =-\mu \zeta \frac{\theta }{\theta_{2}\theta_{1}}dt$$

which satisfies the conditions of Lemma 3. There are P,Q≥0 independent of *t* such that $E∥\theta \left(t\right)∥\leq Qe^{-Pt^{\left(1-2\beta \right)/2}}$.

It is assumed that the subgroup 1,2,⋯,l−1 satisfies the lemma condition, where 3≤l−1≤k. We obtain constants $P_{1},Q_{1}\geq 0$ such that

(24) $$\max_{1\leq i,j\leq l-1}E∥\theta_{i}-\theta_{j}∥\leq Q_{1}e^{-P_{1}t^{\left(1-2\beta \right)/2}}$$

We need to prove the subgroup 1,2,⋯,l also satisfies the lemma condition. Consider the average temperature of all leaders of individual *l*

(25) $${\bar{\theta }}_{l}=\frac{1}{l-1}\sum_{i\in L\left(l\right)}\theta_{i}$$

For each individual j(1≤j≤l−1), using the above equation, we get

(26) $$E∥\theta_{j}-{\bar{\theta }}_{l}∥=\frac{1}{l-1}\sum_{i\in L\left(l\right)}E∥\theta_{j}-\theta_{i}∥\leq Q_{1}e^{-P_{1}t^{\left(1-2\beta \right)/2}}$$

Let $\theta =\theta_{l}-{\bar{\theta }}_{l}$, we get

(27) $$\begin{array}{cc} d\theta & =d\theta_{l}-d{\bar{\theta }}_{l}\\ & =\mu \sum_{k\in L\left(l\right)}\zeta_{lj}\left(\frac{1}{\theta_{l}}-\frac{1}{\theta_{j}}\right)dt-d{\bar{\theta }}_{l}\\ & =-\mu \sum_{j\in L\left(l\right)}\zeta_{lj}\left(\frac{1}{l-1}\sum_{i\in L\left(l\right)}\frac{1}{\theta_{i}}-\frac{1}{\theta_{l}}\right)dt\\ & +\mu \sum_{j\in L\left(l\right)}\zeta_{lj}\left(\frac{1}{l-1}\sum_{i\in L\left(l\right)}\frac{1}{\theta_{i}}-\frac{1}{\theta_{j}}\right)dt-\frac{\mu }{l-1}\sum_{i\in L\left(l\right)}\sum_{j\in L\left(i\right)}\zeta_{ij}\left(\frac{1}{\theta_{i}}-\frac{1}{\theta_{j}}\right)dt\\ & :=-\mu \sum_{j\in L\left(l\right)}\zeta_{lj}\left(\frac{1}{l-1}\sum_{i\in L\left(l\right)}\frac{1}{\theta_{i}}-\frac{1}{\theta_{l}}\right)dt+\xi dt \end{array}$$

The following inequality is given by $\zeta_{lj}<1$ and $\zeta =\sum_{j\in L\left(l\right)}\zeta_{lj}\leq l-1\leq N-1$

(28) $$\begin{array}{cc} E∥\xi ∥ & \leq \mu \sum_{j\in L\left(l\right)}∥\frac{1}{l-1}\sum_{i\in L\left(l\right)}\frac{1}{\theta_{i}}-\frac{1}{\theta_{j}}∥+\frac{\mu }{l-1}\sum_{i\in L\left(l\right)}\sum_{j\in L\left(i\right)}∥\frac{1}{\theta_{i}}-\frac{1}{\theta_{j}}∥\\ & \leq \frac{\mu }{{\left(\theta_{m}^{0}\right)}^{2}}\left(l-1\right)qe^{-pt}+\frac{\mu }{{\left(\theta_{m}^{0}\right)}^{2}}\left(l-1\right)qe^{-pt}=\frac{2\mu }{{\left(\theta_{m}^{0}\right)}^{2}}\left(l-1\right)qe^{-pt} \end{array}$$

The inequality above satisfies Lemma 3. There exist $Q_{2},P_{2}$ such that

(29) $$E∥\theta ∥=E∥\theta_{l}-{\bar{\theta }}_{l}∥\leq Q_{2}e^{-P_{2}\left(1-2\beta \right)/2}$$

Using the norm inequality, we get

(30) $$\begin{array}{cc} E∥\theta_{j}-\theta_{l}∥ & \leq E∥\theta_{j}-{\bar{\theta }}_{l}∥+E∥\theta_{l}-{\bar{\theta }}_{l}∥\\ & \leq Q_{1}e^{-P_{1}\left(1-2\beta \right)/2}+Q_{2}e^{-P_{2}\left(1-2\beta \right)/2}\leq Qe^{-P\left(1-2\beta \right)/2} \end{array}$$

where $Q=Q_{1}+Q_{2},P=\min\left(P_{1},P_{2}\right)$. It is true that

(31) $$\lim_{t\to \infty }E∥\theta_{i}-\theta_{j}∥=0$$

□

### 3.2. Proof of Theorem 2

**Lemma** **4.** *We assume $x,v\in R^{d}$ (considering a hierarchical population of two individuals corresponding to $x_{2}-x_{1},v_{2}-v_{1}$), satisfying the following equations*

(32) $$\begin{array}{cc} dx & =v\\ dv & =\lambda \phi \left(\frac{v_{1}}{\theta_{1}}-\frac{v_{2}}{\theta_{2}}\right)dt-\sigma \phi vdW+\xi_{1}dt+\xi_{2}dW \end{array}$$

*including $\phi \left(∥x∥\right)\in R,\sigma \in R$*

(1)
$1\geq \phi \left(x,t\right)={\left(1+{∥x\left(t\right)∥}^{2}\right)}^{-\beta }>0$

(2)
$E∥\xi_{1}∥,E∥\xi_{2}∥\leq qe^{-pt}\left(p,q>0\right)$

(3)
$\sigma <\sqrt{\frac{2\lambda }{\theta_{M}^{0}}-1}$

*There are P,Q>0, for all t>0, bringing $E∥v\left(t\right)∥\leq Qe^{-Pt^{\left(1-2\beta \right)/2}}$ into existence.*

**Proof.**  We use the $\mathrm{It}\hat{o}$ formula

(33) $$\begin{array}{cc} dv^{2} & =2vdv+dv\cdot dv\\ & =\left(2\lambda \phi \left(v_{2}-v_{1}\right)\cdot \left(\frac{v_{1}}{\theta_{1}}-\frac{v_{2}}{\theta_{2}}\right)+\sigma^{2}\phi^{2}v^{2}\right)dt+\left(2v\xi_{1}-\sigma \phi v\xi_{2}+\xi_{2}^{2}\right)dt\\ & +\left(2\xi_{2}v-2\sigma \phi v^{2}\right)dW\\ & :=\left(L_{1}+L_{2}\right)dt+L_{3}dt+\left(2\xi_{2}v-2\sigma \phi v^{2}\right)dW \end{array}$$

Then, we estimate $L_{1}$

(34) $$\frac{v_{1}}{\theta_{1}}-\frac{v_{2}}{\theta_{2}}=\frac{v_{1}-v_{2}}{\theta_{1}}+v_{2}\left(\frac{1}{\theta_{1}}-\frac{1}{\theta_{2}}\right)=\frac{v_{1}-v_{2}}{\theta_{2}}+v_{1}\left(\frac{1}{\theta_{1}}-\frac{1}{\theta_{2}}\right)$$

(35) $$\begin{array}{cc} L_{1} & =2\lambda \phi \left(v_{2}-v_{1}\right)\cdot \left(\frac{v_{1}-v_{2}}{\theta_{1}}+v_{2}\left(\frac{1}{\theta_{1}}-\frac{1}{\theta_{2}}\right)\right):=L_{11}+L_{12}\\ & =2\lambda \phi \left(v_{2}-v_{1}\right)\cdot \left(\frac{v_{1}-v_{2}}{\theta_{2}}+v_{1}\left(\frac{1}{\theta_{1}}-\frac{1}{\theta_{2}}\right)\right):=L_{13}+L_{14} \end{array}$$

(36) $$\begin{array}{cc} L_{11} & =2\lambda \phi \left(v_{2}-v_{1}\right)\cdot \left(\frac{v_{1}-v_{2}}{\theta_{1}}\right)=-2\lambda \phi \frac{{\left(v_{2}-v_{1}\right)}^{2}}{\theta_{1}}=-\frac{2\lambda \phi }{\theta_{1}}v^{2}\\ L_{12} & =2\lambda \phi \left(v_{2}-v_{1}\right)\cdot v_{2}\left(\frac{1}{\theta_{1}}-\frac{1}{\theta_{2}}\right)=2\lambda \phi \frac{\theta_{2}-\theta_{1}}{\theta_{1}\theta_{2}}\left(v_{2}-v_{1}\right)\cdot v_{2}\\ L_{13} & =2\lambda \phi \left(v_{2}-v_{1}\right)\cdot \left(\frac{v_{1}-v_{2}}{\theta_{2}}\right)=-2\lambda \phi \frac{{\left(v_{2}-v_{1}\right)}^{2}}{\theta_{2}}=-\frac{2\lambda \phi }{\theta_{2}}v^{2}\\ L_{14} & =2\lambda \phi \left(v_{2}-v_{1}\right)\cdot v_{1}\left(\frac{1}{\theta_{1}}-\frac{1}{\theta_{2}}\right)=2\lambda \phi \frac{\theta_{2}-\theta_{1}}{\theta_{1}\theta_{2}}\left(v_{2}-v_{1}\right)\cdot v_{1} \end{array}$$

The calculation results are in the following equations.

(37) $$dv^{2}=\frac{L_{11}+L_{13}}{2}dt+\frac{L_{12}+L_{14}}{2}dt+L_{2}dt+L_{3}dt+\left(2\xi_{2}v-2\sigma \phi v^{2}\right)dW$$

First, we estimate $\frac{L_{11}+L_{13}}{2}$. For $\theta_{1},\theta_{2}\leq \theta_{M}^{0}$

(38) $$\frac{L_{11}+L_{13}}{2}=-\lambda \phi \left(\frac{1}{\theta_{1}}+\frac{1}{\theta_{2}}\right)v^{2}\leq -\frac{2\lambda \phi }{\theta_{M}^{0}}v^{2}$$

Second, we estimate $\frac{L_{12}+L_{14}}{2}$

(39) $$\begin{array}{cc} \frac{L_{12}+L_{14}}{2} & =\lambda \phi \frac{\theta_{2}-\theta_{1}}{\theta_{1}\theta_{2}}\left(v_{2}-v_{1}\right)\cdot v_{2}+\lambda \phi \frac{\theta_{2}-\theta_{1}}{\theta_{1}\theta_{2}}\left(v_{2}-v_{1}\right)\cdot v_{1}\\ & =\lambda \phi \frac{\theta_{2}-\theta_{1}}{\theta_{1}\theta_{2}}v\cdot \left(v_{1}+v_{2}\right) \end{array}$$

From Lemma 3, we have $E∥\theta_{2}-\theta_{1}∥\leq Qe^{-Pt^{\left(1-2\beta \right)/2}}$. From Lemma 2, we have $E∥v_{i}∥\leq D\left(i=1,2\right)$. For $\phi \leq 1,\theta_{m}^{0}\leq \theta_{1},\theta_{2}\leq \theta_{M}^{0}$,

(40) $$\begin{array}{cc} E∥\frac{L_{12}+L_{14}}{2}∥ & =E∥\lambda \phi \frac{\theta_{2}-\theta_{1}}{\theta_{1}\theta_{2}}v\cdot \left(v_{1}+v_{2}\right)∥\\ & \leq \frac{\lambda }{\theta_{1}\theta_{2}}E\left(\phi \right)E∥v\cdot \left(v_{1}+v_{2}\right)∥E∥\theta_{2}-\theta_{1}∥\\ & \leq me^{-nt} \end{array}$$

Third, we estimate $L_{3}$. From Lemma 3.1, there is a nonnegative constant $D_{0}$ causing $E∥v∥\leq D_{0}$. For $E\left(\phi \right)\leq 1$, we take advantage of the properties of expectations,

(41) $$\begin{array}{cc} 2E\left(v\xi_{1}\right)-\sigma E\left(\phi v\xi_{2}\right)+E\left(\xi_{2}^{2}\right) & \leq 2E∥v∥E∥\xi_{1}∥+\sigma E\left(\phi \right)E∥v∥E∥\xi_{2}∥+E\left({∥\xi_{2}∥}^{2}\right)\\ & \leq 2D_{0}qe^{-pt}+\sigma D_{0}qe^{-pt}+q^{2}e^{-2pt}\\ & \leq ce^{-pt} \end{array}$$

where $c=2D_{0}q+\sigma D_{0}q+q^{2}$.

For $\phi \left(t\right)<1$, there are $\phi^{2}\left(t\right)<\phi \left(t\right)$ and $E\left[\phi^{2}\left(t\right)\right]<E\left[\phi \left(t\right)\right]$. For $\sigma <\sqrt{\frac{2\lambda }{\theta_{M}^{0}}-1}$

(42) $$\begin{array}{cc} E\left(-\frac{2\lambda \phi }{\theta_{M}^{0}}+\sigma^{2}\phi^{2}\right) & =-\frac{2\lambda }{\theta_{M}^{0}}E\left(\phi \right)+\sigma^{2}E\left(\phi^{2}\right)\\ & <-\frac{2\lambda }{\theta_{M}^{0}}E\left(\phi \right)+\sigma^{2}E\left(\phi \right)\\ & <-E\left(\phi \right)<-mt^{-2\beta }:=-a\left(t\right) \end{array}$$

From (37), (38), (40), (41) and (42), by defining

(43) $$dV=\left(-a\left(t\right)V+me^{-nt}+ce^{-pt}\right)dt+\left(2V^{\frac{1}{2}}\xi_{2}-2\sigma \phi V\right)dW$$

we obtain

(44) $$\begin{array}{cc} E\left(V\right) & =e^{-\int a\left(t\right)dt}\left(\int \left(me^{-nt}+ce^{-pt}\right)e^{\int a\left(t\right)dt}dt+C\right)\\ & =v^{2}\left(0\right)e^{-\frac{m}{1-2\beta }t^{1-2\beta }}\left(\int \left(me^{-nt}+ce^{-pt}\right)e^{\frac{m}{1-2\beta }t^{1-2\beta }}dt+1\right) \end{array}$$

There are P,Q satisfying $E\left(V\right)\leq Qe^{-Pt^{1-2\beta }}$. According to the comparison theorem, when t>T,

(45) $$\lim_{t\to \infty }E\left(v^{2}\right)\leq \lim_{t\to \infty }E\left(V\right)\leq Qe^{-Pt^{1-2\beta }}$$

□

Next, we give the proof of Theorem 2.

**Proof.**  (Proof of Theorem 2)

For the subgroup $\left\{1,2,\cdots ,l\right\}\left(l=1,2,\cdots ,N\right)$, the theorem is proved by mathematical induction.

The conclusion is proved to be valid for a subpopulation $\left\{1,2\right\}$. According to the definition of the hierarchical group, $L\left(2\right)=1,\phi_{21}>0$, and a constant-speed movement of agent1, we have

(46) $$\begin{array}{c} dx_{1}=v_{1}dt,dx_{2}=v_{2}dt\\ dv_{1}=0,dv_{2}=\lambda \phi_{21}\left(\frac{v_{1}}{\theta_{1}}-\frac{v_{2}}{\theta_{2}}\right)dt+\sigma \phi_{21}\left(v_{1}-v_{2}\right)dW \end{array}$$

We consider

(47) $$x=x_{2}-x_{1},v=v_{2}-v_{1}$$

It is available that

(48) $$\begin{array}{cc} dx & =vdt\\ dv & =\lambda \phi \left(\frac{v_{1}}{\theta_{1}}-\frac{v_{2}}{\theta_{2}}\right)dt-\sigma \phi vdW \end{array}$$

which satisfy the conditions of Lemma 4. The existence of P,Q≥0 independent of *t* make $E∥v\left(t\right)∥\leq Qe^{-Pt^{\left(1-2\beta \right)/2}}$ true.

It is assumed that the subgroup 1,2,⋯,l−1 satisfies the lemma condition, where 3≤l−1≤k. Then we obtain constants $P_{1},Q_{1}\geq 0$ such that

(49) $$\max_{1\leq i,j\leq l-1}E∥v_{i}-v_{j}∥\leq Q_{1}e^{-P_{1}t^{\left(1-2\beta \right)/2}}$$

It is proved that the subgroup 1,2,⋯,l also satisfies the lemma condition. We consider the average position and average velocity of all leaders of individual *l*.

(50) $$\bar{x_{l}}=\frac{1}{l-1}\sum_{i\in L\left(l\right)}x_{i},\bar{v_{l}}=\frac{1}{l-1}\sum_{i\in L\left(l\right)}v_{i}$$

For each individual j(1≤j≤l−1), using the above equation, we get

(51) $$E∥v_{j}-{\bar{v}}_{l}∥=\frac{1}{l-1}\sum_{i\in L\left(l\right)}E∥v_{j}-v_{i}∥\leq Q_{1}e^{-P_{1}t^{\left(1-2\beta \right)/2}}$$

Let $v=v_{l}-\bar{v_{l}},x=x_{l}-\bar{x_{l}}$, we get

(52) $$\begin{array}{cc} dx & =vdt\\ dv & =dv_{l}-d{\bar{v}}_{l}\\ & =\lambda \sum_{j\in L\left(l\right)}\phi_{lj}\left(\frac{v_{j}}{\theta_{j}}-\frac{v_{l}}{\theta_{l}}\right)dt+\sigma_{i}\sum_{j\in L\left(l\right)}\phi_{lj}\left(v_{j}-v_{l}\right)dW_{l}^{t}-d{\bar{v}}_{l}\\ & =-\lambda \sum_{j\in L\left(l\right)}\phi_{lj}\left(\frac{v_{l}}{\theta_{l}}-\frac{1}{l-1}\sum_{i\in L\left(l\right)}\frac{v_{i}}{\theta_{i}}\right)dt-\sigma_{l}\phi_{lj}\left(v_{l}-{\bar{v}}_{l}\right)dW_{l}^{t}\\ & +\lambda \sum_{j\in L\left(l\right)}\phi_{lj}\left(\frac{v_{j}}{\theta_{j}}-\frac{1}{l-1}\sum_{i\in L\left(l\right)}\frac{v_{i}}{\theta_{i}}\right)dt+\sigma_{l}\sum_{j\in L\left(l\right)}\phi_{lj}\left(v_{j}-{\bar{v}}_{l}\right)dW_{l}^{t}\\ & -\frac{\lambda }{l-1}\sum_{i\in L\left(l\right)}\sum_{j\in L\left(i\right)}\phi_{ij}\left(\frac{v_{j}}{\theta_{j}}-\frac{v_{i}}{\theta_{i}}\right)dt-\frac{1}{l-1}\sum_{i\in L\left(l\right)}\sum_{j\in L\left(i\right)}\sigma_{i}\phi_{ij}\left(v_{j}-v_{i}\right)dW_{i}^{t}\\ & :=-\lambda \sum_{j\in L\left(l\right)}\phi_{lj}\left(\frac{v_{l}}{\theta_{l}}-\frac{1}{l-1}\sum_{i\in L\left(l\right)}\frac{v_{i}}{\theta_{i}}\right)dt-\sigma_{l}\phi_{lj}vdW_{l}^{t}+\xi_{1}dt+\xi_{2}dW_{l}^{t} \end{array}$$

From Lemma 2, $E∥v_{i}\left(t\right)∥\leq D$, then we have

(53) $$\begin{array}{cc} ∥\frac{v_{i}}{\theta_{i}}-\frac{v_{j}}{\theta_{j}}∥ & =∥\frac{v_{i}-v_{j}}{\theta_{i}}+v_{j}\left(\frac{1}{\theta_{i}}-\frac{1}{\theta_{j}}\right)∥\\ & \leq ∥\frac{v_{i}-v_{j}}{\theta_{i}}∥+∥v_{j}∥∥\frac{\theta_{i}-\theta_{j}}{\theta_{i}\theta_{j}}∥\leq \frac{1}{\theta_{m}^{0}}∥v_{i}-v_{j}∥+\frac{D}{{\left(\theta_{m}^{0}\right)}^{2}}∥\theta_{i}-\theta_{j}∥ \end{array}$$

It is given by $\phi_{lj}<1,\phi =\sum_{j\in L\left(l\right)}\phi_{lj}\leq l-1\leq N-1$, (49) and temperature consistency that

(54) $$\begin{array}{cc} E∥\xi_{1}∥ & \leq \lambda \sum_{j\in L\left(l\right)}E∥\frac{v_{j}}{\theta_{j}}-\frac{1}{l-1}\sum_{i\in L\left(l\right)}\frac{v_{i}}{\theta_{i}}∥+\frac{\lambda }{l-1}\sum_{i\in L\left(l\right)}\sum_{j\in L\left(i\right)}E∥\frac{v_{j}}{\theta_{j}}-\frac{v_{i}}{\theta_{i}}∥\\ & \leq \frac{\lambda }{\theta_{m}^{0}}\left(l-1\right)qe^{-pt}+\frac{D}{{\left(\theta_{m}^{0}\right)}^{2}}\left(l-1\right)qe^{-pt}+\frac{\lambda }{\theta_{m}^{0}}\left(l-1\right)qe^{-pt}+\frac{D}{{\left(\theta_{m}^{0}\right)}^{2}}\left(l-1\right)qe^{-pt}\\ & =\left(\frac{2\lambda }{\theta_{m}^{0}}+\frac{2D}{{\left(\theta_{m}^{0}\right)}^{2}}\right)\left(l-1\right)qe^{-pt} \end{array}$$

(55) $$\begin{array}{cc} E∥\xi_{2}∥ & \leq \sigma_{l}\sum_{j\in L\left(l\right)}E∥v_{j}-{\bar{v}}_{l}∥+\frac{\sigma }{l-1}\sum_{i\in L\left(l\right)}\sum_{j\in L\left(i\right)}E∥v_{j}-v_{i}∥\\ & \leq \sigma_{l}\left(l-1\right)qe^{-pt}+\sigma \left(l-1\right)qe^{-pt}=2\sigma \left(l-1\right)qe^{-pt} \end{array}$$

The inequality above satisfies Lemma 4 condition. There exist $Q_{2},P_{2}$ such that

(56) $$E∥v∥=E∥v_{l}-{\bar{v}}_{l}∥\leq Q_{2}e^{-P_{2}\left(1-2\beta \right)/2}$$

Using the norm inequality, we get

(57) $$\begin{array}{cc} E∥v_{j}-v_{l}∥ & \leq E∥v_{j}-{\bar{v}}_{l}∥+E∥v_{l}-{\bar{v}}_{l}∥\\ & \leq Q_{1}e^{-P_{1}\left(1-2\beta \right)/2}+Q_{2}e^{-P_{2}\left(1-2\beta \right)/2}\leq Qe^{-P\left(1-2\beta \right)/2} \end{array}$$

where $Q=Q_{1}+Q_{2},P=\min\left(P_{1},P_{2}\right)$. It is true that

(58) $$\begin{array}{cc} \lim_{t\to \infty }E∥v_{i}-v_{j}∥ & =0\\ \underset{1\leq t<\infty }{\sup}E∥x_{i}-x_{j}∥ & =\underset{1\leq t<\infty }{\sup}E∥x_{i}\left(0\right)-x_{j}\left(0\right)+\int_{0}^{t}\left(v_{i}-v_{j}\right)ds∥\\ & \leq ∥x_{i}\left(0\right)-x_{j}\left(0\right)∥+\int_{0}^{t}E∥v_{i}-v_{j}∥ds\leq \infty \end{array}$$

□

## 4. Result Proof of System (3)

**Lemma** **5.** *Let $\left(x_{i},v_{i},\theta_{i}\right)$ be the solution of System (3), where*

(59) $$\int_{0}^{\infty }∥f_{i}\left(t\right)∥dt<\infty$$

*If particle 1 is the global leader and the change in velocity only depends on $f_{1}$, for any t>0, $E∥v_{i}\left(t\right)∥\leq D\left(1\leq i\leq N\right)$, where D>0 is a constant.*

**Proof.**  For particle 1,

(60) $$dv_{1}=f_{1}dt$$

By integrating (60) and computing the expectation, it yields

(61) $$E∥v_{1}∥=\int_{0}^{\infty }∥f_{i}\left(t\right)∥dt<\infty$$

The result holds.

First, we proof $E∥v_{2}\left(t\right)∥$ is bounded by reduction to absurdity. If $E∥v_{2}\left(t\right)∥$ is unbounded, there exists a component $j\left(1\leq j\leq d\right)$ which satisfies $\lim_{t\to \infty }Ev_{2}^{j}\left(t\right)=\infty$. Integrate the second equation of the system and calculate the expected result

(62) $$v_{2}^{j}\left(0\right)=Ev_{2}^{j}\left(t\right)+E\int_{0}^{t}∥f_{2}\left(t\right)∥ds+\lambda \int_{0}^{t}E\phi_{21}\left(\frac{v_{1}^{j}\left(s\right)}{\theta_{1}\left(s\right)}-\frac{v_{2}^{j}\left(s\right)}{\theta_{2}\left(s\right)}\right)\mathrm{ds}$$

Since $\theta_{\min}<\theta_{2}\left(s\right),\theta_{1}\left(s\right)<\theta_{\max},E∥v_{1}∥<\infty$ and $E\int_{0}^{t}∥f_{2}\left(t\right)∥ds<\infty$, (62) implies $v_{2}^{j}\left(0\right)=\infty$. A contradiction appears, which means $E∥v_{2}\left(t\right)∥$ is bounded.

We assume that $E∥v_{2}\left(t\right)∥,\cdots ,E∥v_{l-1}\left(t\right)∥\left(l-1\geq 3\right)$ are bounded. The presence of D≥0 makes $\max_{1\leq i\leq l-1}∥v_{i}\left(t\right)∥\leq D$. A mathematical induction method is used to prove $E∥v_{l}\left(t\right)∥$ is bounded.

Assuming that $E∥v_{l}\left(t\right)∥$ is unbounded, there exists a component $j\left(1\leq j\leq d\right)$, which satisfies $\lim_{t\to \infty }Ev_{l}^{j}\left(t\right)=\infty$. Integrate the equation and calculate the expected result

(63) $$v_{l}^{j}\left(0\right)=Ev_{l}^{j}\left(t\right)+E\int_{0}^{t}∥f_{l}\left(t\right)∥ds+\lambda \int_{0}^{t}\sum_{k\in L\left(l\right)}E\phi_{lk}\left(\frac{v_{l}^{j}\left(s\right)}{\theta_{l}\left(s\right)}-\frac{v_{k}^{j}\left(s\right)}{\theta_{k}\left(s\right)}\right)\mathrm{ds}$$

Since $\theta_{\min}<\theta_{l}\left(s\right),\theta_{k}\left(s\right)<\theta_{\max}$ and $E\int_{0}^{t}∥f_{l}\left(t\right)∥ds<\infty$, (63) implies $v_{l}^{j}\left(0\right)=\infty$. A contradiction appears, which means $E∥v_{l}\left(t\right)∥$ is bounded. □

Since the norm inequality $E∥v_{i}-v_{j}∥\leq E∥v_{i}∥+E∥v_{j}∥$, there is a $D_{0}$ satisfying $E∥v_{i}-v_{j}∥\leq D_{0}$.

From Lemma 5, the proof of the temperature boundedness and consistency of the corresponding system are same as Lemma 3. Next, we discuss the fluctuation of velocities in the corresponding system.

**Lemma** **6.** *We assume $x,v\in R^{d}$ (considering a hierarchical population of two individuals corresponding to $x_{2}-x_{1},v_{2}-v_{1}$), satisfying the following equations*

(64) $$\begin{array}{cc} dx & =v\\ dv & =\lambda \phi \left(\frac{v_{1}}{\theta_{1}}-\frac{v_{2}}{\theta_{2}}\right)dt-\sigma \phi vdW+\left(f_{1}-f_{2}\right)dt+\xi_{1}dt+\xi_{2}dW \end{array}$$

*including $\phi \left(∥x∥\right)\in R,\sigma \in R$*

(1)
$1\geq \phi \left(x,t\right)={\left(1+{∥x\left(t\right)∥}^{2}\right)}^{-\beta }>0$

(2)
$E∥\xi_{1}∥,E∥\xi_{2}∥,E∥f_{1}-f_{2}∥\leq qe^{-pt}\left(p,q>0\right)$

(3)
$\sigma <\sqrt{\frac{2\lambda }{\theta_{M}^{0}}-1}$

*There are P,Q>0, for all t>0, bringing $E∥v\left(t\right)∥\leq Qe^{-Pt^{\left(1-2\beta \right)/2}}$ into existence.*

**Proof.**  We use the $\mathrm{It}\hat{o}$ formula

(65) $$\begin{array}{cc} dv^{2} & =2vdv+dv\cdot dv\\ & =\left(2\lambda \phi \left(v_{2}-v_{1}\right)\cdot \left(\frac{v_{1}}{\theta_{1}}-\frac{v_{2}}{\theta_{2}}\right)+\sigma^{2}\phi^{2}v^{2}\right)dt+\left(2v\left(f_{1}-f_{2}\right)+2v\xi_{1}-\sigma \phi v\xi_{2}+\xi_{2}^{2}\right)dt\\ & +\left(2\xi_{2}v-2\sigma \phi v^{2}\right)dW\\ & :=\left(L_{1}+L_{2}\right)dt+L_{3}dt++\left(2\xi_{2}v-2\sigma \phi v^{2}\right)dW \end{array}$$

The process of estimating $L_{1}$ and $L_{2}$ is the same as Lemma 4. Only the calculation of $L_{3}$ is explained here. From Lemma 5, there is a nonnegative constant $D_{0}$ causing $E∥v∥\leq D_{0}$. For $E\left(\phi \right)\leq 1$, we take advantage of the properties of expectations,

(66) $$\begin{array}{cc} & 2E\left(v\left(f_{1}-f_{2}\right)\right)+2E\left(v\xi_{1}\right)-\sigma E\left(\phi v\xi_{2}\right)+E\left(\xi_{2}^{2}\right)\\ & \leq 2E∥vs.∥E∥f_{1}-f_{2}∥+2E∥v∥E∥\xi_{1}∥+\sigma E\left(\phi \right)E∥v∥E∥\xi_{2}∥+E\left({∥\xi_{2}∥}^{2}\right)\\ & \leq 2D_{0}qe^{-pt}+2D_{0}qe^{-pt}+\sigma D_{0}qe^{-pt}+q^{2}e^{-2pt}\\ & \leq ce^{-pt} \end{array}$$

where $c=4D_{0}q+\sigma D_{0}q+q^{2}$.

It can be seen that (66) is an extension of (41) and thus all subsequent proofs apply. Then we have

(67) $$\lim_{t\to \infty }E\left(v^{2}\right)\leq \lim_{t\to \infty }E\left(V\right)\leq Qe^{-Pt^{1-2\beta }}$$

□

Next, we give the proof of Theorem 3.

**Proof.**  (Proof of Theorem 3)

For the subgroup $\left\{1,2,\cdots ,l\right\}\left(l=1,2,\cdots ,N\right)$, the theorem is proved by mathematical induction.

The conclusion is proved to be valid for a subpopulation $\left\{1,2\right\}$. According to the definition of hierarchical group, $L\left(2\right)=1,\phi_{21}>0$, and a constant-speed movement of agent1, we have

(68) $$dx_{1}=v_{1}dt,dx_{2}=v_{2}dt$$

(69) $$dv_{1}=f_{1}dt,dv_{2}=\lambda \phi_{21}\left(\frac{v_{1}}{\theta_{1}}-\frac{v_{2}}{\theta_{2}}\right)dt+f_{2}dt+\sigma \phi_{21}\left(v_{1}-v_{2}\right)dW$$

We consider

(70) $$x=x_{2}-x_{1},v=v_{2}-v_{1}$$

It is available that

(71) $$\begin{array}{cc} dx & =vdt\\ dv & =\lambda \phi \left(\frac{v_{1}}{\theta_{1}}+\left(f_{2}-f_{1}\right)dt-\frac{v_{2}}{\theta_{2}}\right)dt-\sigma \phi vdW \end{array}$$

which satisfy the conditions of Lemma 6. The existence of P,Q≥0 independent of *t* make $E∥v\left(t\right)∥\leq Qe^{-Pt^{\left(1-2\beta \right)/2}}$ true.

It is assumed that the subgroup 1,2,⋯,l−1 satisfies the lemma condition, where 3≤l−1≤k. Then we obtain constants $P_{1},Q_{1}\geq 0$ such that

(72) $$\max_{1\leq i,j\leq l-1}E∥v_{i}-v_{j}∥\leq Q_{1}e^{-P_{1}t^{\left(1-2\beta \right)/2}}$$

It is proved that the subgroup 1,2,⋯,l also satisfies the lemma condition. We consider the average position and average velocity of all leaders of individual *l*.

(73) $$\bar{x_{l}}=\frac{1}{l-1}\sum_{i\in L\left(l\right)}x_{i},\bar{v_{l}}=\frac{1}{l-1}\sum_{i\in L\left(l\right)}v_{i}$$

For each individual j(1≤j≤l−1), using the above equation, we get

(74) $$E∥v_{j}-{\bar{v}}_{l}∥=\frac{1}{l-1}\sum_{i\in L\left(l\right)}E∥v_{j}-v_{i}∥\leq Q_{1}e^{-P_{1}t^{\left(1-2\beta \right)/2}}$$

Let $v=v_{l}-\bar{v_{l}},x=x_{l}-\bar{x_{l}}$, we get

(75) $$\begin{array}{cc} dx & =vdt\\ dv & =dv_{l}-d{\bar{v}}_{l}\\ & =\lambda \sum_{j\in L\left(l\right)}\phi_{lj}\left(\frac{v_{j}}{\theta_{j}}-\frac{v_{l}}{\theta_{l}}\right)dt+\sigma_{i}\sum_{j\in L\left(l\right)}\phi_{lj}\left(v_{j}-v_{l}\right)dW_{l}^{t}-d{\bar{v}}_{l}\\ & =-\lambda \sum_{j\in L\left(l\right)}\phi_{lj}\left(\frac{v_{l}}{\theta_{l}}-\frac{1}{l-1}\sum_{i\in L\left(l\right)}\frac{v_{i}}{\theta_{i}}\right)dt-\sigma_{l}\phi_{lj}\left(v_{l}-{\bar{v}}_{l}\right)dW_{l}^{t}+\left(f_{l}-\frac{1}{l-1}\sum_{i\in L\left(l\right)}f_{i}\right)dt\\ & +\lambda \sum_{j\in L\left(l\right)}\phi_{lj}\left(\frac{v_{j}}{\theta_{j}}-\frac{1}{l-1}\sum_{i\in L\left(l\right)}\frac{v_{i}}{\theta_{i}}\right)dt+\sigma_{l}\sum_{j\in L\left(l\right)}\phi_{lj}\left(v_{j}-{\bar{v}}_{l}\right)dW_{l}^{t}\\ & -\frac{\lambda }{l-1}\sum_{i\in L\left(l\right)}\sum_{j\in L\left(i\right)}\phi_{ij}\left(\frac{v_{j}}{\theta_{j}}-\frac{v_{i}}{\theta_{i}}\right)dt-\frac{1}{l-1}\sum_{i\in L\left(l\right)}\sum_{j\in L\left(i\right)}\sigma_{i}\phi_{ij}\left(v_{j}-v_{i}\right)dW_{i}^{t}\\ & :=-\lambda \sum_{j\in L\left(l\right)}\phi_{lj}\left(\frac{v_{l}}{\theta_{l}}-\frac{1}{l-1}\sum_{i\in L\left(l\right)}\frac{v_{i}}{\theta_{i}}\right)dt-\sigma_{l}\phi_{lj}vdW_{l}^{t}+\xi_{1}dt+\xi_{2}dW_{l}^{t} \end{array}$$

From Lemma 5, $E∥v_{i}\left(t\right)∥\leq D$, then we have

(76) $$\begin{array}{cc} ∥\frac{v_{i}}{\theta_{i}}-\frac{v_{j}}{\theta_{j}}∥ & =∥\frac{v_{i}-v_{j}}{\theta_{i}}+v_{j}\left(\frac{1}{\theta_{i}}-\frac{1}{\theta_{j}}\right)∥\\ & \leq ∥\frac{v_{i}-v_{j}}{\theta_{i}}∥+∥v_{j}∥∥\frac{\theta_{i}-\theta_{j}}{\theta_{i}\theta_{j}}∥\leq \frac{1}{\theta_{m}^{0}}∥v_{i}-v_{j}∥+\frac{D}{{\left(\theta_{m}^{0}\right)}^{2}}∥\theta_{i}-\theta_{j}∥ \end{array}$$

It is given by $\phi_{lj}<1,\phi =\sum_{j\in L\left(l\right)}\phi_{lj}\leq l-1\leq N-1$, (72) and temperature consistency that

(77) $$E∥f_{l}-\frac{1}{l-1}\sum_{i\in L\left(l\right)}f_{i}∥\leq \left(l-1\right)qe^{-pt}$$

(78) $$\begin{array}{cc} E∥\xi_{1}∥ & \leq \lambda \sum_{j\in L\left(l\right)}E∥\frac{v_{j}}{\theta_{j}}-\frac{1}{l-1}\sum_{i\in L\left(l\right)}\frac{v_{i}}{\theta_{i}}∥+\frac{\lambda }{l-1}\sum_{i\in L\left(l\right)}\sum_{j\in L\left(i\right)}E∥\frac{v_{j}}{\theta_{j}}-\frac{v_{i}}{\theta_{i}}∥\\ & \leq \frac{\lambda }{\theta_{m}^{0}}\left(l-1\right)qe^{-pt}+\frac{D}{{\left(\theta_{m}^{0}\right)}^{2}}\left(l-1\right)qe^{-pt}+\frac{\lambda }{\theta_{m}^{0}}\left(l-1\right)qe^{-pt}+\frac{D}{{\left(\theta_{m}^{0}\right)}^{2}}\left(l-1\right)qe^{-pt}\\ & =\left(\frac{2\lambda }{\theta_{m}^{0}}+\frac{2D}{{\left(\theta_{m}^{0}\right)}^{2}}\right)\left(l-1\right)qe^{-pt} \end{array}$$

(79) $$\begin{array}{cc} E∥\xi_{2}∥ & \leq \sigma_{l}\sum_{j\in L\left(l\right)}E∥v_{j}-{\bar{v}}_{l}∥+\frac{\sigma }{l-1}\sum_{i\in L\left(l\right)}\sum_{j\in L\left(i\right)}E∥v_{j}-v_{i}∥\\ & \leq \sigma_{l}\left(l-1\right)qe^{-pt}+\sigma \left(l-1\right)qe^{-pt}=2\sigma \left(l-1\right)qe^{-pt} \end{array}$$

The inequality above satisfies Lemma 6. There exist $Q_{2},P_{2}$ such that

(80) $$E∥v∥=E∥v_{l}-{\bar{v}}_{l}∥\leq Q_{2}e^{-P_{2}\left(1-2\beta \right)/2}$$

Using the norm inequality, we get

(81) $$\begin{array}{cc} E∥v_{j}-v_{l}∥ & \leq E∥v_{j}-{\bar{v}}_{l}∥+E∥v_{l}-{\bar{v}}_{l}∥\\ & \leq Q_{1}e^{-P_{1}\left(1-2\beta \right)/2}+Q_{2}e^{-P_{2}\left(1-2\beta \right)/2}\leq Qe^{-P\left(1-2\beta \right)/2} \end{array}$$

where $Q=Q_{1}+Q_{2},P=\min\left(P_{1},P_{2}\right)$. It is true that

(82) $$\begin{array}{cc} \lim_{t\to \infty }E∥v_{i}-v_{j}∥ & =0\\ \underset{1\leq t<\infty }{\sup}E∥x_{i}-x_{j}∥ & =\underset{1\leq t<\infty }{\sup}E∥x_{i}\left(0\right)-x_{j}\left(0\right)+\int_{0}^{t}\left(v_{i}-v_{j}\right)ds∥\\ & \leq ∥x_{i}\left(0\right)-x_{j}\left(0\right)∥+\int_{0}^{t}E∥v_{i}-v_{j}∥ds\leq \infty \end{array}$$

□

## 5. Numerical Simulation

**Figure 1:**
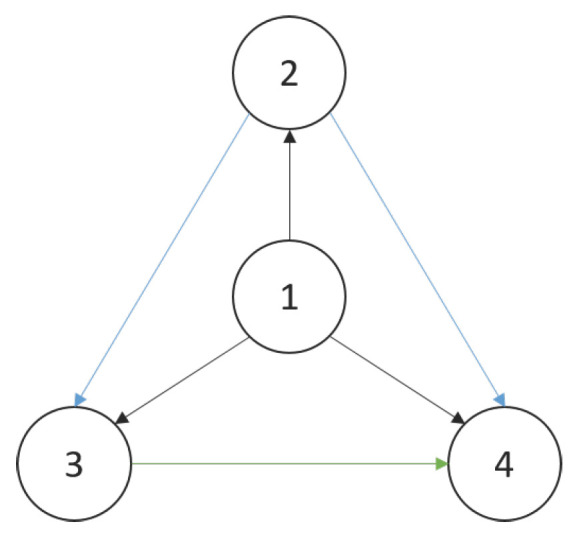
Connectivity topology under hierarchical leadership for 4 individuals.

This section uses computer simulation to verify the results of the theorems. Figure 1 shows the directed graph of the hierarchy in the numerical experiment with four individuals as examples. The arrow in the figure points from the leader to the follower.

The total time *t* is 50 s, λ is 10 and μ is 5. For the initial data, we consider 12 particles as the hierarchical group whose initial positions and velocities are random numbers uniformly distributed in the interval $\left[-1,1\right]$.

### 5.1. Numerical Experiments for System (2)

We take σ=1 and β=0.5, which satisfies the theorem condition of this paper. Simulation results verify the correctness of the conclusion.

**Figure 2:**
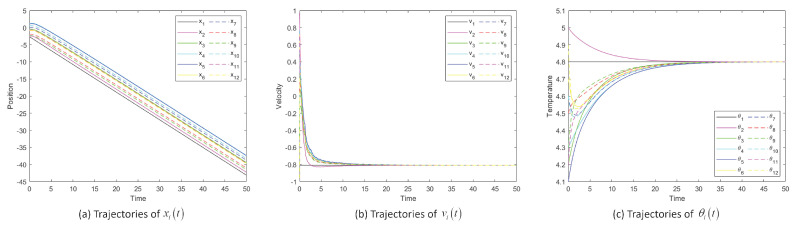
Trajectories of position $x_{i}\left(t\right)$, velocity $v_{i}\left(t\right)$ and temperature $\theta_{i}\left(t\right)$ of System (2) and flocking behavior for σ=1.

Figure 2 shows the position, velocity and temperature trajectory images of System (2) changing with time when noise intensity σ is 1. The temperature and velocity of each particle tend to match that of the overall leader (particle 1).

**Figure 3:**
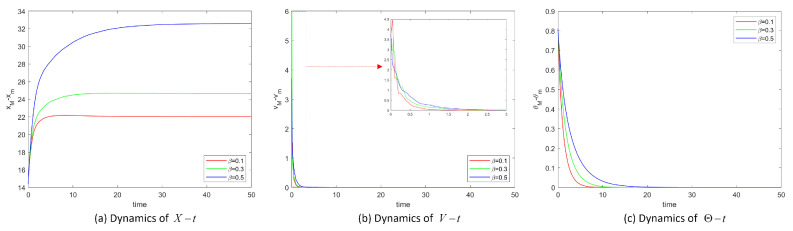
Position difference $x_{M}-x_{m}$, velocity difference $v_{M}-v_{m}$ and temperature difference $\theta_{M}-\theta_{m}$ of System (2) for σ=1.

Figure 3 shows the position difference, velocity difference and temperature difference images of System (2) when noise intensity σ is 1. We introduce some functionals to describe the flocking process: $X=x_{M}-x_{m}$, $V=v_{M}-v_{m}$ and $\Theta =\theta_{M}-\theta_{m}$, which mean the differences between the maximum and minimum values at moment *t*. Figure 3a, b and c display the dynamics of X−t, V−t and Θ−t showing the fluctuations around the overall leader as a function of the choice of the coupling strength. Taking the same initial values, the systems converge when the values of β are 0.5, 0.3 and 0.1. The lower the β value, the larger the communication coefficient, and the sooner the system will reach flocking.

**Figure 4:**
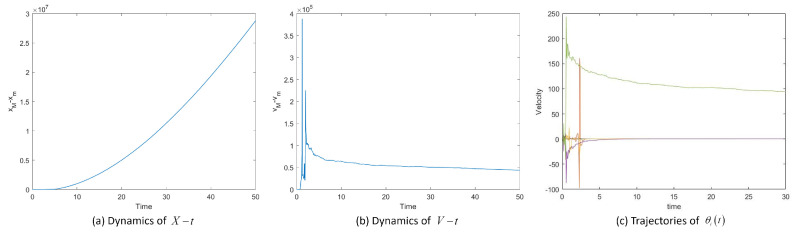
Position difference *X*, velocity difference *V* and velocity trajectories $v_{i}\left(t\right)$ of System (2) with σ=5.

Then we take σ=5 and β=0.5. Figure 4a and b, show the dynamics of X−t, V−t and Figure 4c shows the velocity trajectory of each particle. System (2) is unable to reach flocking state if the noise value is too large to satisfy the condition of the theorem. There is a tolerance limit to noise.

As shown in Figure 2, Figure 3 and Figure 4, when certain conditions are met, the positions, velocities and temperatures in System (2) are globally flocking, which shows the correctness of Theorem 1 and Theorem 2. The simulation results agree well with the conditions of our theorem, thus verifying our theorem. Due to the chaotic nature of the system, there is some oscillation in the velocity or velocity difference of the system.

### 5.2. Numerical Experiments for System (3)

For System (3), the disturbance functions are as follows.

$$f_{1}=\frac{1}{{\left(1+t\right)}^{2}};f_{i}=\frac{1}{{\left(1+t\right)}^{2}}+ae^{-bt},a>0,b>0,\left(i\neq 1\right)$$

We take σ=1, which satisfies the theorem condition of this paper. Simulation results verify the correctness of the conclusion.

**Figure 5:**
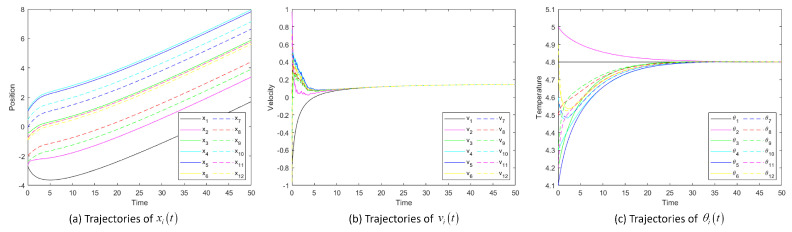
Trajectories of position $x_{i}\left(t\right)$, velocity $v_{i}\left(t\right)$ and temperature $\theta_{i}\left(t\right)$ of System (3) and flocking behavior for σ=1.

Figure 5 shows the position, velocity and temperature trajectory images of System (3) changing with time when noise intensity σ is 1 and β is 0.3. Figure 5a, b and c show the states of all particles attaining flocking. After a few fluctuations, the particle swarm moves forward in a fixed formation around the leader (particle 1). Each particle’s temperature and velocity tend to match particle 1. This shows that the system achieves flocking, which is consistent with the description of the theorem.

**Figure 6:**
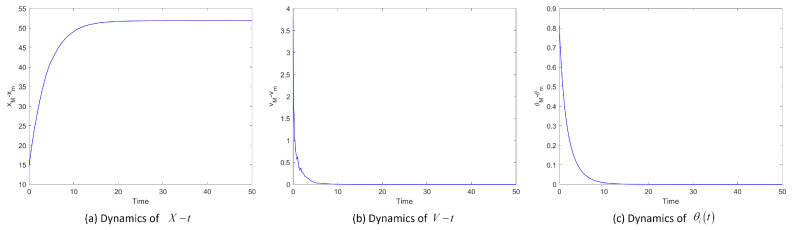
Position difference $x_{M}-x_{m}$, velocity difference $v_{M}-v_{m}$ and temperature difference $\theta_{M}-\theta_{m}$ of System (3) for σ=1.

In Figure 6, corresponding to σ=2, Figure 6a, b and c show the position difference X−t, velocity difference V−t and temperature difference Θ−t images of System (3). We observe that the experimental data are discrete and that particles move with a degree of freedom; the velocity curve is not smooth. Velocity difference tends to 0 over time; the system reaches flocking. For the TCS system, it has a noise term in the differential equation for velocity. The fluctuation of velocity decays exponentially.

**Figure 7:**
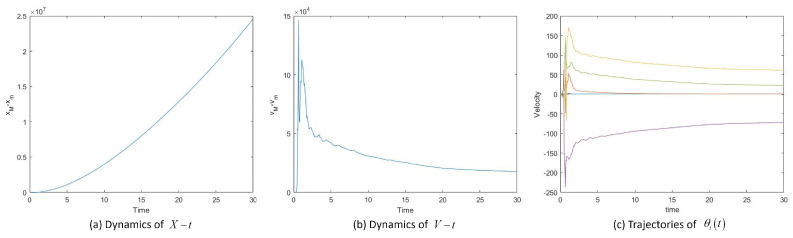
Position difference $x_{M}-x_{m}$, velocity difference $v_{M}-v_{m}$ and velocity trajectories $v_{i}\left(t\right)$ of System (3) with σ=5.

Then we take σ=5. Figure 7a and b show the dynamics of X−t, V−t and Figure 7c shows the velocity trajectory of each particle in System (3). If the noise value is too large to satisfy the conditions of the theorem, the system will not reach the flocking state.

As shown in Figure 5, Figure 6 and Figure 7, when certain conditions are met, the positions, velocities and temperatures in System (3) are globally flocking, which shows the correctness of Theorem 3. The above numerical experiments show that in a TCS system with white noise under hierarchical leadership, adding a small perturbation to the velocity variation, the system can still achieve flocking when the noise satisfies the theorem conditions.

## 6. Conclusions

In this paper, the effect of multiplicative white noise on a TCS system with free disturbance under a hierarchical system is considered. Due to the properties of hierarchy, the system can be studied by mathematical induction. It is proved that, when the noise intensity meets certain conditions, the system can achieve flocking. The results are verified by numerical simulation. Similarly, we study the above models with perturbation functions and generate good results. We show that particles can keep pace with the determined overall leader, even if there exist some noises. Small perturbations do not affect the convergence of the hierarchy up to a certain noise limit.

Based on this system, other factors such as communication delay, system chaos and individual collision avoidance are all problems to be solved. According to the actual situation, this model is established to study the evolution of flocking dynamics. This mechanism can solve the problem of system consistency and cooperation, and has considerable application value in multi-robot flocking cooperation, UAV formation control, etc. For example, when the robot queue is disturbed, the whole team can also be consistent with the leader.

## Data Availability

No new data were created.

## Abbreviations

The following abbreviations are used in this manuscript:

CS	Cucker–Smale
TCS	Thermodynamic Cucker–Smale
UAV	Unmanned aerial vehicle
